# Record linkage based patient intersection cardinality for rare disease studies using Mainzelliste and secure multi-party computation

**DOI:** 10.1186/s12967-022-03671-6

**Published:** 2022-10-08

**Authors:** Tobias Kussel, Torben Brenner, Galina Tremper, Josef Schepers, Martin Lablans, Kay Hamacher

**Affiliations:** 1 Technische Universität Darmstadt, Schnittspahnstraße 10, 64287 Darmstadt, Germany; 2 German Cancer Research Center, Im Neuenheimer Feld 580, 69120 Heidelberg, Germany; 3 University Medical Centre Mannheim, Theodor-Kutzer-Ufer 1-3, 68167 Mannheim, Germany; 4 Berlin Institute of Health, Anna-Louisa-Karsch-Str. 2, 10178 Berlin, Germany

**Keywords:** Multi-party computation, Rare disease, Intersection cardinality, Record linkage, Medical informatics

## Abstract

**Background:**

The low number of patients suffering from any given rare diseases poses a difficult problem for medical research: With the exception of some specialized biobanks and disease registries, potential study participants’ information are disjoint and distributed over many medical institutions. Whenever some of those facilities are in close proximity, a significant overlap of patients can reasonably be expected, further complicating statistical study feasibility assessments and data gathering. Due to the sensitive nature of medical records and identifying data, data transfer and joint computations are often forbidden by law or associated with prohibitive amounts of effort. To alleviate this problem and to support rare disease research, we developed the Mainzelliste Secure EpiLinker (MainSEL) record linkage framework, a secure Multi-Party Computation based application using trusted-third-party-less cryptographic protocols to perform privacy-preserving record linkage with high security guarantees. In this work, we extend MainSEL to allow the record linkage based calculation of the number of common patients between institutions. This allows privacy-preserving statistical feasibility estimations for further analyses and data consolidation. Additionally, we created easy to deploy software packages using microservice containerization and continuous deployment/continuous integration. We performed tests with medical researchers using MainSEL in real-world medical IT environments, using synthetic patient data.

**Results:**

We show that MainSEL achieves practical runtimes, performing 10 000 comparisons in approximately 5 minutes. Our approach proved to be feasible in a wide range of network settings and use cases. The “lessons learned” from the real-world testing show the need to explicitly support and document the usage and deployment for both analysis pipeline integration and researcher driven ad-hoc analysis use cases, thus clarifying the wide applicability of our software. MainSEL is freely available under: https://github.com/medicalinformatics/MainSEL

**Conclusions:**

MainSEL performs well in real-world settings and is a useful tool not only for rare disease research, but medical research in general. It achieves practical runtimes, improved security guarantees compared to existing solutions, and is simple to deploy in strict clinical IT environments. Based on the “lessons learned” from the real-word testing, we hope to enable a wide range of medical researchers to meet their needs and requirements using modern privacy-preserving technologies.

## Background

Rare Diseases affect 5% of the German population[5] and, albeit individually rare, constitute a major cost driver in health care. One challenge in medical research targeting those diseases is the availability of sufficient amounts of data to extract statistically significant insight with respect to causes and treatments. Strict data protection laws relating to medical and identifiable personal data further increase the difficulty of the task, especially when data of affected patients is spread across many health care facilities.

Before conducting clinical trials with data from different clinic sites, it is usually necessary to determine the patient numbers that can be pooled horizontally. If the patient numbers at all participating sites are very small—for example when dealing with rare diseases—and if even the sum of the numbers from several sites is in a critical range for the significance of the results, it makes sense to validate in advance that no patients were included twice or even more often in the analyses. This can occur, for example, if patients are registered not only at one but at several sites—a common problem in rare disease research due to diagnostic challenges. Before taking further steps in study planning, it is desirable to know that the intersection of different sites is empty or at least very small.

Conversely, when planning vertical data pooling—for example, when considering linking differentiated but monotemporal patient data from hospitals with longitudinal subject history data from registries—it is desirable to have as large an intersection of documented subjects as possible.

In both scenarios, challenges pertaining to data transfer are a common occurrence. According to Article 6 of the EU General Data Protection Regulation (GDPR), the transfer of data from one data owner to another or a common database is generally prohibited as long as no consent has been obtained from the people described by the data.

To protect the sensitive patient care data, it should be stored separately from the medical records, which are used for research. In this way, a researcher can use the medical data without knowing the patient identity. Traditionally, both types of records are linked by pseudonyms, which allow assigning additional medical data to the existing patient. To join distributed medical records, they need first to be linked to unique patients. In an ideal world, this could be realized using a trans-institutional “master patient index”, containing a unique identifier for each unique patient. However, this unique id is an identifying field in itself and is subject to the same data privacy considerations as the other identifying data fields. Privacy-preserving techniques for distributed computations can be—and are being—used to utilize unique identifies for set operations, e.g. [41]. In some countries, existing identifiers such as the patient’s health insurance number could be used as a unique identifier. In Germany, however, less than 90% of patients make use of a statutory health insurance [19] and are thus issued such an insurance id. A solution that would nonetheless allow the linkage, would be to use record linkage methods based on the full set of identifiable patient data, without disclosing those data.

Established record linkage implementations (e.g., [25, 34]) are mostly based on Privacy-Preserving Record Linkage (PPRL) techniques using pre-processed (e.g., hashed) identifying data such as first and surname, birth date, address, etc. that is linked centrally using a Third Trusted Party (TTP) [50]. As with any centralized approach, this can turn out to be a problem if the TTP is compromised or becomes untrustworthy. Additionally, centralized Bloom filter-based systems are known to be vulnerable against frequency and cryptanalysis attacks [8, 51]. Even though recent versions claim protection against those known attacks [46], new exploits are expected to be found [55]. Alternatives to centralized PPRL are Record Linkage systems based on secure Multi-Party Computation (MPC) [18, 35, 48] which allow participating parties to perform a joined linkage calculation over distributed input data without the need of any central linkage component. In particular, by ensuring that the data is not transferred, thus never leaving its owner’s institutional boundaries, the attack surface of the identifying data is reduced, increasing the patients’ data protection level and prohibiting potential re-identification. While the legal assessment is still pending, the possibility of using MPC for some distributed computations *without explicit patient consent* is worth entertaining. Error-tolerant record linkage has the advantage over “classical” Private Set Intersection (PSI), as it allows “fuzzy” matches, increasing the linkage quality on noisy, incomplete data.

In this paper, we describe our method for determining record linkage based patient intersection using MPC, as well as its evaluation under the real clinical conditions within the Collaboration on Rare Diseases project (CORD_MI)^1^. The joint research project CORD_MI is a use case which involves four German medical informatics consortia, 24 German clinical centers and partner organizations, and multiple patient associations. Funded by the German Federal Ministry of Education and Research (BMBF), the main aim of the CORD project is to improve care and research in the field of rare diseases. We show that our novel method is feasible in real world medical contexts of rare disease research, in particular within the IT environments of nine German university hospitals. We provide an open-source, easy to deploy, holistic record linkage, intersection cardinality, pseudonymization and ID management system. Finally, we discuss the “lessons learned” while deploying our system in multiple medical institutions.

### Related work

Related to the problem addressed in this work are Private Set Intersection Cardinality (PSI-CA) Protocols [10, 28, 31]. However, while being highly efficient and optimized, PSI and PSI-CA protocols are only suited to find *exact* intersections. This is a constraint, that renders them unsuitable for clinical applications. Patients’ identifying data (IDAT) are subject to change, e.g., due to relocation, marriages, and errors.

The methods performing this “fuzzy” matching between patient records are called *Record Linkage* methods (RL) [14], and specifically in the context of this work *Privacy-Preserving Record Linkage* (PPRL) [15, 35]. For a more detailed discussion of related works in the field of Record linkage, please confer [18, 48], and for an overview of PPRL methods, including a discussion w.r.t. cryptographic privacy and statistical perturbation, please see [24].

## Methods

The aim of our research is to define and test a method for finding patient record intersections based on record linkage for data distributed over multiple medical data holders.

For this task it is necessary to consider the following real world constraints:

- The sensitive patient data is to be protected, no information allowing the re-identification of the patient should leave the data owner’s location.
- For security reasons no trusted party should be used for record linkage. This condition will also simplify the patient consent process and, thus, allow recruiting more patients for the research for rare diseases.
- Even in the case of an IT security incident in one of the participating party’s protected networks, the patients’ data of all other institutions must remain private.
- The proposed method assures high precision and coverage in determining the intersection set in order to be feasible for sparse data in the field of rare diseases.

Today’s Bloom filter-based solutions fail to meet the first three requirements (see Sect. ), while non-record linkage based MPC private set intersection algorithms fail the last one. Our approach to tackle these challenges is to design a method for record linkage based patient intersection using full-threshold secure Multi-Party computation.

In the following, we explain the basic concepts of patient list management as well as secure Multi-Party Computation (MPC) that are essential for subsequent parts of this paper.

### Record linkage

The process and techniques to compare two data sources (often comprising medical patient’s identifying data) to link duplicates is called *record linkage* (RL). For many medical applications and medical research it is either a first step in the analysis, or even the analysis itself (e.g., determining the patient intersection count, or the total number of unique patients in two or more data sources). Formally, the process can be seen as composed of two distinct steps: 1. calculating the similarity between two records, and 2. classifying those records as duplicate (“match”) or independent patients (“non match”) [14].

*The EpiLink algorithm.* This work is based on the *EpiLink* algorithm [9], as implemented in the Mainzelliste pseudonymization framework [33] for local de-duplication. This algorithm further subdivides the first step above, the calculation of a similarity measure, in two steps. First, a similarity is calculated comparing each field in the record (e.g., how similar are the birthdays). Those field similarities are then combined to a total patient similarity. This combination is a (normalized) weighted sum, allowing to differentiate the “importance” of the individual fields, according to statistical factors. Both the individual field similarities and the total patient similarities are represented as real numbers between zero (completely different) and one (identical).

In step 2, the classification, this patient similarity is compared to two configurable thresholds. If the similarity is below the first threshold, the two patients are considered distinct and thus classified as non matches. If the similarity is above the *second* threshold, the two patients are classified as a very likely match. If it is between the two thresholds, the records are marked as a *tentative match*.

Furthermore, the Mainzelliste RL algorithm, as well as our algorithm, uses *exchange groups*, a configurable number of fields, where all permutations are compared, e.g. first, sur- and birth name. These fields may be easily swapped during data entry in error. For more details on our implementation of exchange groups, see [48].

*Field comparisons.* To further increase the flexibility and quality of the record linkage, and in fact allowing the required fault-tolerant “fuzzy” matching, the EpiLink algorithm allows every field similarity measure that produces real numbers between zero and one.

MainSEL implements two field comparison operations, one checking the identity of an entry, e.g., the year of birth, and a “fuzzy” string comparison. The system can be extended with other specialized comparison functions, for example an edit distance comparison for genomic data [49].

*Fuzzy string comparison.* For “fuzzy” field comparisons, the fields content (namely, strings) are converted to *Bloom filters* [4]. The similarity measure is the Dice-coefficient [12] of both parties’ Bloom filters. In the following sections, we will introduce and explain these concepts in more detail.

**Fig. 1:**
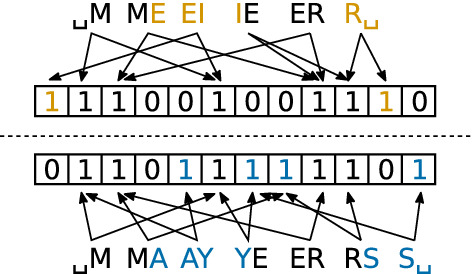
Visual example of the Bloom filter-based Dice similarity measure. In this example, the strings “MEIER” and “MAYERS” are compared, using $k=2$ different hash functions and a 12 bit bloom filter. The colors mark the differences. Note, that a change of one letter leads to at most 2*k* different set bits, that is, small changes in the strings lead to small changes in the bit pattern

The *Bloom filter Dice similarity* introduced by Schnell et al [47] starts by converting the input data into Bloom filters, a probabilistic data structure often used in database design to quickly estimate whether an element is likely included in a database. The input data are separated into a list of *n-grams*, in practice often *bigrams*, i.e., groups of two characters (cf. Fig. 1). Each *n*-gram is hashed with *k* independent hash functions, resulting in *k* numbers. These numbers are then converted to indices for a bitfield with *m* bits by using the modulo operation. On those positions, the bits in the bitfield are set to one.

Our system uses those Bloom filters in an atypical way, not for a fast element lookup and not as a privacy measure, as in other PPRL systems, including [47], but only as a data structure. As it can be seen in the example in Fig. 1, the division in *n*-grams yield Bloom filters, where small changes result in only a small change of bits (at most *k* bit per different *n*-gram).

The similarity measure used is the *Dice coefficient*. It is calculated by counting the number of elements, where in both Bloom filters the bit is set to one. This number is multiplied by two and divided by the total number of the set bits in both filters:

1 $$\begin{aligned} S&= \dfrac{2{\text {Hw}} ({\text {Bl}} (x)\wedge {\text {Bl}} (y))}{{\text {Hw}} ({\text {Bl}} (x))+{\text {Hw}} ({\text {Bl}} (y))}, \end{aligned}$$

where *x* and *y* are the inputs, ${\text {Bl}}$ the function to transform the inputs into a Bloom filter, ${\text {Hw}}$ the *Hamming Weight* operation [22] (counting the set bits), $\wedge$ the element-wise logical “and” operation and, finally, *S* the resulting similarity score between zero and one.

For a recent overview over PPRL methods and tools, see Gkoulalas-Divanis et al [18].

### Secure multi-party computation

*Secure Multi-Party Computation* (MPC) is a cryptographic technique to jointly compute a function over distributed inputs, without leaking any more information about the parties’ inputs than the result of the computation reveals. Being a research area within the academic field of cryptography, MPC ’s privacy guarantee are mathematically provable [20, 37].

The field of MPC was started by Andrew Yao’s seminal work “How to Generate and Exchange Secrets” in 1986 [54]. This work introduced the “Yao’s Garbled Circuits” two-party protocol. One year later Goldreich et al published the multiparty GMW protocol [20], named after its authors. Even though the theoretical groundwork thus were laid in the mid 80s, MPC only achieved practical performance and usability with the publication of the first MPC compiler, Fairplay [38], in 2004. Since then, advancements in computation hardware, novel protocol optimizations, such as Half Gates [56], Oblivious Transfer Extensions [1], or free-XOR [30], and completely new protocols (e.g., [29]) allowed practical run times for more and more complex use cases. Today, applications ranging from secure contact discovery on mobile devices [28] to genomic analysis [49], epidemiological modeling [21], and privacy-preserving machine learning [40] have been published. However, even with the availability of a number of programming frameworks assisting the development of MPC applications, achieving good—or at least acceptable—run times remains a difficult task requiring cryptographic expertise.

In the following paragraphs, we briefly describe the three two-party protocols used in this work. For a more detailed overview, survey article and textbooks should be consulted, e.g., [23, 36].

*Yao’s Garbled Circuits.* Yao’s Garbled Circuit (GC) [54] operates on *Boolean Circuits*, i.e., the function that is to be jointly computed must be represented in a directed acyclic graph with the logic operations “and” ($\wedge$) and “xor” ($\oplus$) as nodes. Following the naming convention of electrical engineering, the nodes—containing the operations—are called *gates* and the edges, containing bit-wise values, are called *wires*. This representation, the algebraic normal form, can represent arbitrary bounded functions.

Both parties assume different roles in the protocol, namely the *garbler* and the *evaluator*. The garbler constructs the Boolean circuit and *garbles* it by assigning every possible bit value on every wire in the circuit a random AES key. The truth tables of the gates (i.e., the input-to-output mapping) are generated by doubly encrypting the output keys with the corresponding two input keys. The order is then permuted. After the garbling, only the garbler can translate any key to a corresponding logical value. The circuit construction and the garbling is independent of any party’s specific input and can be prepared in the *setup phase*.

This garbled circuit and the keys encoding the garbler’s input values are then transferred to the evaluator. This first interaction between both parties marks the start of the *online phase*. The evaluator receives the keys encoding his secret inputs with a cryptographic primitive called *Oblivious Transfer* (OT) [43, 52]. Using OT the evaluator receives his keys without learning any other key and the garbler learns nothing regarding the evaluator’s input. The evaluator proceeds to evaluate the circuit in a gate-by-gate fashion. The final output keys are then translated by the garbler and distributed to both parties. A modern formal description, as well as the full security proof, can be found in [37].

Using Yao’s GC, the required communication scales with the *and-size* of the circuit, that means the number of and-gates in the circuit. As it is based on Boolean Circuits, it is especially efficient in evaluating bitwise operations, comparisons, and branching by multiplexing values based on decision bits.

*GMW.* The GMW protocol [20] uses Boolean circuits as well and works by breaking up the secret input into multiple fragments, called *shares*, such that the secret can only be reconstructed if a party holds *all* shares. The secret sharing is information theoretically secure, i.e., even an adversary with infinite time and unbound computation power cannot break the system. The secret input bits $v_i$ are broken into *n* shares each, which are then one-by-one sent to the $n-1$ other parties. Note, that by this *full-threshold* construction, even if all other parties collude, nobody holds *all* shares of the secret and the secret inputs remain private. For each secret bit, the shares $s_i$ are generated in the following way: The first $n-1$ shares are just random bits, independently drawn from a (cryptographically secure) uniform distribution. The last share $s_n$ is generated by xor-ing all previous shares, as well as the secret bit *v*. Or more formally:

$$\begin{aligned} s_i&\leftarrow _{\$},\quad i \in \{1, 2, \dots , n-1\},\\ s_n&\leftarrow \bigoplus _i s_i \oplus v. \end{aligned}$$

After the distribution of the secrets, every party holds one share of every secret. The reconstruction of a secret is performed by xor-ing all shares together.

The xor operation can be performed locally by every party, simply by xor-ing the shares it holds. This generates a valid share of the result. The and operation is performed in an interactive protocol involving Oblivious Transfer (cf. Section 2.2), thus, requiring additional communication between the participating parties.

Using the GMW protocol, the required communication scales with the *and-depths* of the circuit, that means the number of “layers” of the graph containing and gates. As it is based on Boolean Circuits, it is especially efficient in evaluating bitwise operations, comparisons, and branching by multiplexing values based on decision bits.

*Arithmetic Secret Sharing.* Arithmetic Secret Sharing can be understood as an extension of the GMW protocol on algebraic rings $\mathbb {Z}_k$ of size *k* instead of Boolean values. Thus, it operates on arithmetic circuits, that is, circuits using addition and multiplication operations. Hence, those arithmetic operations are much more efficient to evaluate in this protocol, compared to Boolean circuit-based protocols. The secret sharing process is similar to the GMW secret sharing. The xor operation is substituted with a subtraction on the ring:

$$\begin{aligned} s_i&\leftarrow _{\$}{\mathbb {Z}_{k}},\quad i \in \{1, 2, \dots , n-1\},\\ s_n&\leftarrow v - \sum \limits _i s_i \mod k. \end{aligned}$$

The reconstruction of the secret value *v* works by adding all the shares (modulo *k*). Multiplication require, as in the Boolean protocol variant, an interactive protocol, e.g., using “Beaver’s multiplication triples” [2], or the “Gilboa multiplication” [17].

Both Boolean GMW and Arithmetic Secret Sharing perform most communication and invocations of (symmetric) cryptographic primitives during the setup phase, allowing for a faster online phase.

*ABY Framework.* ABY is an open source, high performance C++ Framework for semi honest, hybrid protocol Secure Two-Party Computation [11]. It allows the construction and evaluation of application using Arithmetic Secret Sharing, Boolean GMW and Yao’s GC, as well as the conversion between the different sharings. This allows a fine-grained optimization of the MPC, as different sections of the calculation might be more efficient in different protocols.

By choosing a rather low abstraction level for developing circuits, ABY trades ease of development with many (manual) low-level optimization possibilities for the protocol design.

For the detailed security guarantees of ABY in general and MainSEL in particular, please see [11, 48].

### Mainzelliste framework for patient pseudonymization

Mainzelliste [33] is a web-based open source pseudonymization software that is actively used to link records between multiple^2^ medical research infrastructures [27, 39, 42], biobanks [3], and patient registries [32].

Mainzelliste can function as a master patient id generator, as well as a primary and secondary pseudonym management tool in combination with identifying data. It can perform local probabilistic record linkage for patient de-duplication using a highly optimized version [45] of the EpiLink [9] algorithm.

In previous work, we modularly extended Mainzelliste with the MPC based trans-institutional PPRL system, *Secure EpiLinker* [48]. The secure EpiLinker is connected to Mainzelliste via its RESTful API, following the general design of Mainzelliste, thus forming a holistic system for pseudonymization, record linkage and ID management: MainSEL (freely available under https://github.com/medicalinformatics/MainSEL).

By using MPC techniques, MainSEL can utilize all identifying information for a robust and accurate fault-tolerant record linkage, while simultaneously providing confidentiality and higher security guarantees than existing solutions.

## Results

### MainSEL: Mainzelliste Secure EpiLinker

In this work, we extended the MainSEL system presented in [48] to allow privacy-preserving intersection cardinality calculations based on the record linkage algorithm. This extension builds upon the versatility of circuit-based MPC protocols by reusing the record linkage similarity calculation and match classification and replacing the id distribution circuit with the set cardinality calculation. An early proof-of-concept was already described in [48], however, the expectation for practical interfaces, additional run time analysis, as well as the full functionality was achieved as part of the work presented here.

As described in previous works (e.g., [34, 48]), the deployment of biomedical applications in clinical networks is complex and expensive, due to non-standard network topologies, high network compartmentalization, and strict regulatory requirements [6, 7]. Usually, those networks have a high security level, due to the sensitive data processed inside of them. One common restriction is the limitation of direct TCP (incoming) connections by firewall systems and proxies. This prevents the communication between the Secure EpiLinker instances, as those use raw TCP sockets for communication to optimize network bandwidth utilization. Furthermore, the bidirectional nature of the necessary communication requires inbound port forwarding rules in the firewall systems, which are in need of extensive monitoring and auditing.

**Fig. 2:**
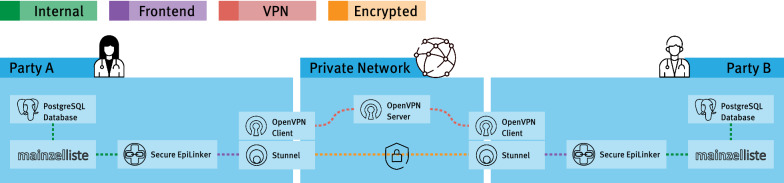
MainSEL architectural overview. The diagram shows two MainSEL Docker Compose stacks, both interacting in a virtual, private network established by a OpenVPN server. Only the OpenVPN and Stunnel components are interfacing to the open network, all MainSEL core components use stack-internal networking

These challenges are solved by the MainSEL system architecture developed in this work using an overlay network (cf. Fig. 2).

*Network Transport Encryption and Authentication.* Some network boundaries only allow traffic to egress through HTTP(S) proxy systems, which often discard any traffic not conforming to those protocols. To enable communication between MainSEL instances despite this restriction, all MainSEL traffic is routed through a “Stunnel” [53]. Stunnel adds TLS encryption to the communication, without modifications to the other components. With that, all network packets conform to the HTTPS structure. As the data payload is encrypted, which is indistinguishable from random data, a proxy cannot inspect the data and thus not discard the non-HTTPS data.

An additional benefit is the easily usable added client/server authentication. No adversary is able to impersonate one of the genuine computation parties. Stunnel allows both public-key cryptography based approaches, as well as pre-shared key (PSK) symmetric schemes.

Note that the transport layer encryption is not required to satisfy the stated privacy and security guarantees.

*Virtual Private Network Aggregation.* Proposed changes to the firewall rules of clinical IT networks are (rightfully) thoroughly screened and evaluated before implementation. This is especially true for rules regarding incoming traffic, as those pose a greater risk for the network to become compromised.

These change processes exist for a good reason and are a necessary element of every production system’s security strategy. However, to allow timely testing in real-world networks using synthetic data, MainSEL was required to solely operate with outgoing dedicated connections.

To achieve this mode of operation, the MainSEL system connects to a virtual private network (VPN) at the beginning of a calculation. The outgoing TCP connection of the VPN client of each party to the VPN server is used for all communication.

Note, that this setup does not weaken any security notion, even though it increases the complexity of the system. The MPC protocol remains secure, even if all clear-text traffic is observed by an adversary [20, 37]. Additionally, MainSEL’s traffic is encrypted by Stunnel (see Section 3.1).

MainSEL uses OpenVPN^3^ as both the VPN client and server. OpenVPN is a well-known open source VPN solution, highly regarded due to its security and simplicity of operation. A full security audit was performed in 2017 [44].

*Containerization and Deployment.* The software components necessary for successfully using MainSEL in restricted network environments, add significant complexity to the building and deployment process. To allow the easy and resource-efficient deployment despite that complexity, a *Docker Compose*^4^ containerization was chosen, tying all components together via two compartmentalized overlay networks, one for backend communication and one for the outside-facing component. Docker Compose allows the connection and orchestration of multiple software modules in lightweight docker^5^ containers with provided sane default configurations. Compared to classical virtual machine deployments, where each virtual machine uses its own operating system and kernel resources, containerization allows only compartmentalizing of the *application* layer. The underlying operating system is shared between all running containers, thus saving (memory) resources.

This deployment schema not only reduces the required setup effort, it greatly enhances the automation possibilities, as well as the reproducibility of our experiments. Using methods from the “Continuous Integration / Continuous Deployment” [13] paradigm, changes to the source code of the components result in an automatic testing and building process. The resulting docker images can be deployed with minimal delay, relieving deploying organizations from the burden of providing a functioning build environment. As a result, version mismatches are unlikely and identical test environments for reproducible experiments can be set up quickly.

The chosen service-based abstraction provides scalability, as based on the expected workload all services can either run on one physical server or be deployed on multiple servers with minimal (if any) changes to the configuration.

### Performance benchmarks

*Test Setup.* We first evaluated our protocol in a lab environment consisting of two virtual servers connected via LAN with a latency of 0.3 ms and a bandwidth of 1 Gbit/s. The servers were equipped with a virtual 6 Core processor and 24 GiB of RAM each. We averaged all benchmarks over 10 independent runs.

We compare three different network models:

**A:** 1 Gbit/s bandwidth and no additionally imposed delays. This setting is relevant as most university medical centers in Germany are connected via the “Deutsches Forschungs-Netz (DFN)” (German research network), a high-bandwidth carrier network dedicated to research. In this context, bandwidths of 1 Gbit/s are a conservative lower bound.
**B:** Restricted throughput (100 Mbit/s), but no imposed latency.
**C:** High latency (100 ms) and restricted bandwidth (100 Mbit/s).

Models B and C are interesting to analyze different protocol variations, namely:

**GMW:** The system is using the GMW without conversion to arithmetic secret sharing during score evaluation.

**Yao:** The system is using Yao’s Garbled Circuits without conversion to arithmetic secret sharing during score evaluation.

**GMW/A:** The system is using the GMW protocol and conversion to arithmetic secret sharing during score evaluation.

**Yao/A:** The system is using Yao’s Garbled Circuits and conversion to arithmetic secret sharing during score evaluation.

In addition to the protocol behavior analysis, the high-latency setting is suitable to better estimate real-world performance, as many security applications in clinical networks, such as packet inspection, proxy systems, and firewalls, introduce an additional delay to the network connection.

All tests are performed using the default Mainzelliste field configuration. This includes the fields first name, surname, birth name, day of birth, month of birth, year of birth, ZIP code, and City. The three name fields form an exchange group. For more information, see [48].

**Fig. 3:**
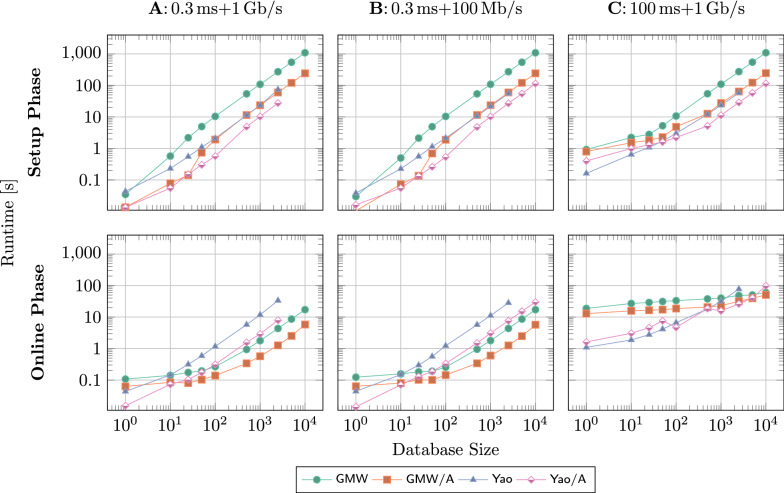
Setup and online runtime in seconds for *varying database sizes* and four circuit variants (cf. Sect. 3.2), in three network environments: **A**: 0.3 ms latency+Gbit/s bandwidth, **B**: 0.3 ms+100 Mbit/s, **C**: 100 ms+ 1 Gbit/s. The field configuration of the Mainzelliste, developed by the German Cancer Research Center (DKF), was used in all benchmarks

*Measurements.* We performed the benchmarks by measuring the comparison of one record against a varying number of records for the second party, across three network models and four protocol configurations. Figure 3 shows the resulting runtimes, grouped in the *Setup Phase*, which consists of the pre-calculations before the inputs are known, and the *Online Phase*, which consists of the actual computation with the real input data. The tests show, that the computation of 10 000 comparisons runs in roughly five minutes considering the worst-case network model and around 20% faster in the best-case network model.

Table 1 shows the runtimes, interaction rounds, and the size of the incurred communication in the **GMW/A** configuration.

**Table 1:** Comparison of the setup and online runtimes of the MPC RL based intersection cardinality procedure of varying numbers of records in circuit variant $\mathsf {GMW/A}$. Compared are the three networking configurations from Figure 3, for *varying database sizes*. The reported network communication cost is the sum of sent and received data

Database	Comm. [MiB]			Setup Phase [s]			Online Phase [s]		
Size	#Rounds	Setup	Online	A	B	C	A	B	C
1	266	0.6	0.1	0.014	0.01	0.8	0.063	0.063	13
10	330	5.7	0.7	0.078	0.073	1.5	0.085	0.081	16
25	346	14.1	1.7	0.14	0.14	1.8	0.081	0.1	16
50	362	28.1	3.4	0.73	0.69	2.3	0.1	0.1	17
100	378	53.7	6.7	1.9	1.9	4.9	0.14	0.14	19
500	410	279	25.6	12	11	13	0.34	0.34	21
1,000	426	557.8	47.1	24	23	28	0.57	0.6	23
2,500	458	1,394.4	115.5	60	60	64	1.3	1.3	32
5,000	474	2,788.6	222.5	120	120	120	2.5	2.5	39
10,000	490	5,577.4	444.9	240	240	250	5.8	5.7	51

### Real world, synthetic data tests

In addition to the performance benchmarks (cf. Section ), we conducted several real-world tests between eight German clinical and university locations (see Table 2). As the legal assessment of using MPC protocols with real patient data is still pending, synthetic datasets were used for these experiments.

The goal of these tests was twofold: first to get feedback from (bio)medical researchers on how well MainSEL solves their requirements, and second to allow researchers to gather experience using MPC applications. This experience is necessary to sway the way MPC is seen in the community, from an experimental, highly academical technique to a well-understood, mature way to solve distributed research needs, especially in the field of rare diseases.

**Table 2:** Pairings of institutions participating in the synthetic data, real world tests

Test Number	Party 1	Party 2	Party 3
1	University Medical Centre Mannheim	RTWH Aachen University	Berlin Institute of Health
2	University Hospital Carl Gustav Carus, Dresden	University Hospital Frankfurt	University Medical Centre Mannheim
3	University Hospital Tübingen	University Hospital Würzburg	University Hospital Regensburg

Helper scripts and pre-setup configuration files were supplied, as well as OpenVPN servers to lower the barrier to conduct the tests. A wide range of feedback and researchers’ background were prioritized over customized deployment scenarios. One key difference between this ad hoc deployment in comparison to the deployment as a part of a permanent research pipeline is the data ingestion. For this test run, MainSEL ingested CSV files as described below.

Mainzelliste, and with that MainSEL, is able to directly integrate into FHIR^6^-based pipelines^7^. HL7 FHIR is an established standard in medical informatics, so the ingestion of FHIR data is highly encouraged for permanent installations.

*Test Data Generation* The synthetic datasets for the evaluation of MainSEL in real-world clinical environments were based on the synthetic FHIR datasets, used by the CORD project to promote interoperability and specialized rare disease documentation (e.g. Orpha code) in German medical facilities. Those datasets were modeled to cover the “MI-I Kerndatensatz” [16] (common-core dataset of the German medical informatics initiative). For the evaluation of MainSEL’s functionalities, the attributes of the module “Person”, describing a patient’s identifiable and demographic data, were sufficient. We extracted around 54 000 Records as *Comma Separated Value* (CSV) tables, i.e., “flattened” clear text tables and increased the variations of the first name, last name, and city name fields by sampling from a list of 50 000 patient and city names created using the mock data generator “Mockaroo”.^8^

**Fig. 4:**
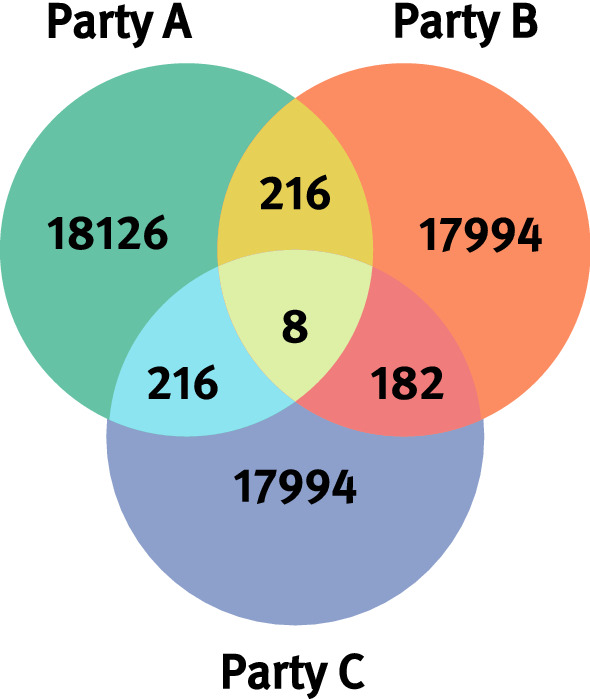
Composition of the synthetic datasets. All three generated datasets consist of roughly 18 000 records. The pairwise overlap consists of around 200 records. In addition, 8 records are included in all three datasets

We prepared three datasets with around 18 000 records each and a known pairwise overlap, as well as a small common overlap between all three datasets (Fig. 4).

**Fig. 5:**
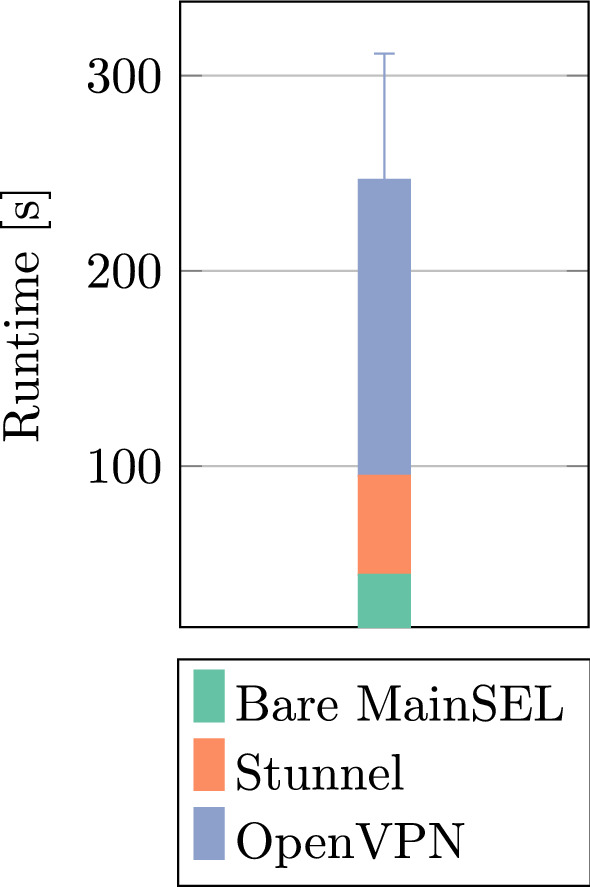
Runtime composition of the full MainSEL system comparing two databases with 100 patients each. The “Bare MainSEL” setup consists of only the PostgreSQL, Mainzelliste, and Secure EpiLinker containers

*Runtime Performance* We measured the runtime of a computation between two parties with 100 patients each using varying system compositions to assess the runtime cost of the additional cryptographic components, namely, the Stunnel and OpenVPN containers (Fig. 5). The system specifications of both parties are the same as in the performance benchmark setup (cf. 3.2). The OpenVPN Server ran on a server using an AMD EPYC™ 7702 processor with 4 dedicated cores running on 3.34 GHz, 16 GiB RAM, and a 2.5 Gbit/s network interface, sufficient to fully saturate the two parties’ bandwidth.

## Discussion

After an initial “transient response” for small databases, the benchmarks (Fig. 3; Table 1) show the expected linear relationship between the number of compared records and overall runtime. It is visible, that the usage of conversions to arithmetic secret shares is beneficial in nearly all scenarios, the exception being high-latency networks with small databases. Additionally, the benefit of the constant-round Yao’s Garbled Circuit protocol over GMW in high-latency networks is visible. This is not surprising, as the delay incurs a runtime penalty for each of the multiple hundred interaction rounds in the GMW protocol.

The network setting is a major influence on the run times. For 10 000 comparisons, the runtime differs more than 20% between the best and the worst network model.

Both, the performance benchmarks, and the real-world test (Fig. 5) attest MainSEL practical, feasible performance for real-world workloads, thus providing a useful tool for medical researchers. However, the measurement of the runtime composition in the real-world setting reveals the high overhead due to the secure networking components. Removing OpenVPN from the stack results in a 259% performance boost, and removing Stunnel results in an additional 214% improvement. The usage of *both* systems results in an approximately 5.6 times worse runtime. This strongly suggests that the exploration of other proxy and firewall traversal mechanisms, as well as the implementation of authenticated communication channels directly in the ABY framework, are fundamental steps to further increase MainSEL’s practicality for larger workloads.

The separation between *Setup Phase* and *Online Phase* is beneficial, as it provides insight for gauging the feasibility of an “online system” mode of operation. With this, we mean a continuously running system updating the intersection count in the background, e.g., after every patient admission. In this case, the setup phase can be run in between admissions with the online phase as the only “observable” delay between data entry and result.

*Lessons Learned from Real World Tests.* All test sites were able to perform the complete test suite with the correct results. Most evaluations were attended by at least one MainSEL developer to solve occurring hindrances promptly and interview the researchers regarding their experiences with MainSEL. The functionality and the stronger security guarantees were well received by the rare disease researchers, indicating the need for secure, pragmatic tools.

One commonly observed difficulty was based on some researchers’ wish to perform the computations by hosting MainSEL on their desktop computers, rather than on dedicated server hardware. Due to SARS-CoV-2 restrictions, most researchers worked remotely, so that residential internet bandwidths vastly increased calculation times beyond realistically feasible durations. When using institutional internet connections, most test cases could be completed within less than ten minutes. This underlines MainSEL’s use case as a permanent element in an institutional research pipeline or for one-off computations, hosted within clinical or research networks. Additionally, real patients’ data – contrary to the used, synthetic data sets—are generally only allowed in restricted network settings, thus eliminating this observed problem by policy.

Most other difficulties were related to either the test-scenario-specific helper scripts or the provided documentation. Some portability issues regarding converting file system paths between the Linux and Microsoft Windows operating systems were discovered and solved. To make things worse, in the test period Docker Desktop on Windows system introduced a major version change of Docker Compose breaking backward compatibility. To conclude this group of obstacles: it became apparent, that our approach of providing easy-to-use scripts for complexity abstraction interfered with the expectations and usage patterns of technically highly skilled researchers. This eventually led to more difficulties compared to *requiring* more detailed configuration from the users.

The last two classes of hindrances were technical in nature, again. We worked with the participating researchers to accommodate the high variety of encountered proxy systems. The specific network configuration led to multiple configuration combinations required for normal operation. We are confident to cover most proxy configurations as a result of those tests, however, different needs cannot be ruled out. Lastly, the OpenVPN configuration used for this test and discussed in Section 3.1—while technically compatible with all institutions’ firewalls—can be misused to circumvent the network policies the firewalls are supposed to implement. This is acceptable for test usage with synthetic data. For real operational readiness, specialized gateways with well-defined functionality and operation concepts coordinated with the institutions’ IT security teams are required.

## Conclusion

In this work, we extended the MainSEL framework for secure, privacy-preserving record linkage to allow the secure calculation of patient set intersection cardinalities. This functionality was highly requested in CORD_MI. Possible use cases include study feasibility estimation and dedicated analyses. In comparison to “classical” Private Set Intersection (PSI) solutions, our solution allows a successful computation on noisy, incomplete, and faulty patient records.

We provided an easy-to-deploy software packet, achieving practical run times in real-world networks. Using the full system, 10 000 patient comparisons can be performed in around 5 min. To assure up-to-date deployments, easy extensibility, and easy integration, we employ the methods of the “Continuous Integration / Continuous Deployment” (CI/CD) paradigm, e.g., automated build and containerization pipelines.

In cooperation with the rare disease research community, we conducted tests in eight German university medical centers using synthetic data. We incorporated this community feedback and worked on simplifying the usage, adaptation to different IT environments, and documentation, as well as training for participating researchers.

A number of promising research directions were identified, thanks to the real-world tests. To achieve reasonable run times for very large databases, computation reduction techniques look promising, but challenging, as some techniques, such as locality sensitive hashing are known to conflict with MPC security models [24]. Furthermore, different proxy and firewall traversal systems, as well as the implementation of authenticated communication channels in the ABY framework would allow a performance improvement of up to 557%. Finally, the extension of the EpiLink record linkage algorithm to allow multi-party similarities, that is, direct record linkage between more than two parties, is a frequently requested feature. This extension is not trivial, due to the non-transitivity of the EpiLink similarity.

Using MPC as the cryptographic basis of MainSEL provides vastly higher security guarantees than existing solutions, e.g. those based on centralized Bloom filters. In particular:

- Sensitive patient data is protected using modern, tried-and-tested cryptographic primitives. Informally speaking, even the “weakest” cryptographic building-block used in MainSEL (OT-Extension) uses state-of-the-art elliptic curve cryptography and is deemed safe until a sufficiently powerful quantum computer is built.
- No trusted third party or central component has to take part in the computation. There is no central collection of sensible identifying data of any kind.
- Even if all but one of the participating parties are compromised, all uncompromised parties’ patient data remain private.

These improved security guarantees have the potential to make MPC based methods the new state of the art in record linkage. In fact, a recent legal investigation [26] argues that MPC techniques of this kind do not constitute data transfer as defined in the European General Data Protection Regulation (GDPR). The individual assessment of each specific implementation, however, is required. While this work was devised on a European legal basis, similar restrictions exist in other jurisdictions. MPC-based techniques might provide similar benefits there as well. In particular, the proposed harmonization of European and United States’ data protection laws in the *Transatlantic Data Privacy Framework* (TADPF), as well as the federal *American Data Privacy and Protection Act* (ADPPA), might thus lead to a direct applicability of this work to U.S.-based research partners. Future legal research will shine more light on this topic. Nonetheless, MPC-based distributed analysis techniques could enable researchers to perform analyses previously not allowed, e.g., rendering the need for a data transfer consent obsolete, leading to easier and quicker study designs. In any case, MPC based record linkage, as used in MainSEL, provides unprecedented privacy levels for patients in rare diseases and beyond.

## Data Availability

The MainSEL software implementation is freely available under an permissive open source license in the MainSEL Github repository, https://github.com/medicalinformatics/MainSEL The datasets used and analysed during the current study are available from the corresponding author on reasonable request.

## Footnotes

1 https://www.medizininformatik-initiative.de/en/CORD.

2 At the time of writing, around 20 projects, not including the authors’ own applications. See https://bitbucket.org/medicalinformatics/mainzelliste/src/development/README.md (accessed 12.03.2022) for details.

3 https://openvpn.net.

4 https://github.com/docker/compose.

5 https://www.docker.com.

6 https://www.hl7.org/fhir/R4.

7 thanks to the open-source contribution of Marcel Parciak of the University Hospital in Göttingen https://bitbucket.org/medicalinformatics/mainzelliste/pull-requests/179 (accessed 12.03.2022).

8 https://mockaroo.com.
